# Exonic Short Interspersed Nuclear Element Insertion in *FAM161A* Is Associated with Autosomal Recessive Progressive Retinal Atrophy in the English Shepherd

**DOI:** 10.3390/genes15070952

**Published:** 2024-07-20

**Authors:** Katherine Stanbury, Ellen C. Schofield, Bryan McLaughlin, Oliver P. Forman, Cathryn S. Mellersh

**Affiliations:** 1 Canine Genetics Centre, Department of Veterinary Medicine, University of Cambridge, Cambridge CB3 0ES, UK; 2 Wisdom Panel, Mars Petcare (Science and Diagnostics Division), Freeby Lane, Waltham on the Wolds, Leicestershire LE14 4RS, UK

**Keywords:** genetics, dogs, progressive retinal atrophy, retinitis pigmentosa, *FAM161A*, inherited eye disease, whole genome sequencing, homozygosity mapping, SINE

## Abstract

Progressive retinal atrophies (PRAs) are a genetically heterogeneous group of inherited eye diseases that affect over 100 breeds of dog. The initial clinical sign is visual impairment in scotopic conditions, as a consequence of rod photoreceptor cell degeneration. Photopic vision degeneration then follows, due to progression of the disease to the cone photoreceptors, and ultimately results in complete blindness. Two full-sibling English Shepherds were diagnosed with PRA at approximately 5 years old and tested clear of all published PRA genetic variants. This study sought to identify the novel PRA-associated variant segregating in the breed. We utilised a combined approach of whole genome sequencing of the probands and homozygosity mapping of four cases and 22 controls and identified a short interspersed nuclear element within an alternatively spliced exon in *FAM161A*. The XP_005626197.1 c.17929_ins210 variant was homozygous in six PRA cases and heterozygous or absent in control dogs, consistent with a recessive mode of inheritance. The insertion is predicted to extend exon 4 by 39 aberrant amino acids followed by an early termination stop codon. PRA is intractable to treatment, so the development of a genetic screening test, based on the associated variant, is significant, because it provides dog breeders/owners with a means of reducing the frequency of the disease variant within this breed as well as minimising the risk of breeding puppies that will develop this blinding disease.

## 1. Introduction

Canine inherited eye diseases (IEDs) affect over 160 breeds of dog and are caused by variants located in over 60 genes [1]. Almost half of these genes cause inherited and progressive disorders specific to the retina, a group of diseases known as progressive retinal atrophies, or PRAs. On ophthalmic examination, PRAs present bilaterally with attenuation of retinal blood vessels, retinal thinning which manifests as increased reflectivity of the tapetal later, and in the latter stages of disease, atrophy of the optic nerve head [2,3]. PRA presents at a variable age of onset between and within dog breeds, depending on disease aetiology. Early-onset forms, such as in the Irish Setter [4] and Norwegian Elkhound [5], are caused by aberrant differentiation of photoreceptors during retinal development. In late-onset PRAs, development of cone and rod photoreceptors is normal; however, differing mechanisms involving the structure, function and maintenance of these cells are impaired [2]. There is considerable overlap of the clinical presentation and physiopathology of canine PRAs with retinitis pigmentosa (RP) in humans [6]. A recent study estimated that a surprisingly high proportion of humans carry IED variants, with a third of the global population estimated to be heterozygous for at least one IED-causing variant [7]. RP is the most common IED within this disease group, with a prevalence of 1:3000–1:4000 in the human population [8]. As with PRA, RP results in blindness for the affected individual and the effects of the disease in both dogs and humans have to date been resistant to medical therapies. Progress in the field of gene therapy to manipulate photoreceptor degeneration is being made [7,8]; however, there is only one approved therapy in humans that is specific to patients with a variant in the *RPE65* gene [9]. However, the number of affected individuals with this variant is low and accounts for only 0.3–1% of RP cases [9]. The canine eye is analogous to the human eye in both size and morphology, but it is the equivalent photoreceptor density in the *area centralis* to the human macula that makes it advantageous as a model for human retinal diseases [10]. Furthermore, the dog, with its unique population structure, can identify novel variants that could shed light on retinal degeneration in other species, including human RP, and potentially identify novel gene therapy targets.

This study sought to identify the variant associated with PRA in the English Shepherd Dog (ESD). The ESD breed purportedly originated from ancient English and Scottish herding dogs and was then established in the United States after early settlers took them across the Atlantic in the 17th century [11]. It is not registered by either the UK Kennel Club or the American Kennel Club but has been registered with the United Kennel Club since 1927 [11]. This project was initiated when two ESD PRA-affected siblings (hereby referred to as probands 1 and 2) tested clear of all previously published canine IED genetic variants. This suggested a novel variant segregating in the breed that was causal for PRA. Pedigree analysis of the probands suggested a recessive mode of inheritance for the disease. We used a combined whole genome sequencing and homozygosity mapping approach which identified an exonic short interspersed nuclear element (SINE) within a strong candidate gene, *FAM161A.*

## 2. Materials and Methods

### 2.1. Sample Collection

ESD DNA samples were collected by dog owners who submitted buccal mucosal swab samples from their dogs to the Canine Genetics Centre (previously based at the Animal Health Trust, Newmarket, UK) (AHT Ethics Approval ref: 24-2018E, University of Cambridge Ethics Approval ref: CR564). The diagnosis of individual dogs as either clear of inherited eye disease or PRA-affected was determined by veterinary ophthalmologists, either by clinical referral or the British Veterinary Association/Kennel Club/International Sheep Dog Society (BVA/KC/ISDS) Eye Scheme or US equivalent. Dogs defined as PRA cases (*n* = 6) were diagnosed after ophthalmic examination with bilateral retinal degeneration, tapetal hyperreflectivity and retinal vascular attenuation. Proband 1, a female ESD, was referred to a veterinary ophthalmologist at the age of 4.83 years after the owner reported visual impairment in dim light. Significant findings from ophthalmic examination included a reduced menace response in scotopic conditions, bilateral observations of generalised tapetal hyper-reflectivity, pallor of the optic nerve head, generalised depigmentation of the non-tapetal fundus with multiple small areas of hyperpigmentation and retinal vascular attenuation. Proband 2, a male sibling to proband 1, was found to be clear of eye disease at age 1.75 years during a routine BVA eye examination. On a subsequent BVA eye examination at age 4.89 years, proband 2 was diagnosed with clinical signs consistent with PRA, including bilateral retinal degeneration, marked tapetal hyperreflectivity and vascular attenuation. Further unrelated cases submitted were between the ages of 4.83 and 8.92 years with a median age of 4.99 years. Control dogs certified clear of inherited eye disease were aged 7 years or above (*n* = 20). The median age of controls was 10 years and the average age was 10.11 years. 

### 2.2. Exclusion of Known Canine Inherited Eye Disease Variants

Probands 1 and 2 were genotyped by gel electrophoresis and massively parallel sequencing using a custom in-house-designed amplicon array panel of PCR amplicons of known canine IED variants (published up to 2019) (Supplementary File S1). 

### 2.3. Whole Genome Sequencing

Once the two probands were confirmed as clear of all published canine IED variants, samples were prepared for whole genome sequencing (WGS). WGS was outsourced to Cancer Research UK, Cambridge Institute, University of Cambridge, where an Illumina 150 bp paired-end DNA library was prepared and sequenced on an Illumina Novaseq 6000, generating approximately 30× genome coverage. Paired-end sequence data were aligned to the CanFam4 UU Cfam GSD 1.0 reference genome with BWA-MEM v0.7 [12]. Base quality score recalibration, indel realignment, duplicate removal and SNP/INDEL discovery were performed using GATK v4.2 [13] according to GATK Best Practices recommendations [14]. Joint-calling was performed across 309 samples, including the two ESD cases, using standard hard filtering parameters or variant quality score recalibration. The filtered variants were annotated and functional effects predicted using SnpEff v5.1 [15].

### 2.4. WGS Variant Filtering

The first stage of variant filtering was performed using WGS of 307 dogs, comprising 109 breeds and 2 cross-breeds, via an in-house pipeline that scores variants based on the predicted effect on the protein. The 307 WGSs consisted of dogs with varying phenotypes, including PRA. However, as all known canine IED variants had already been excluded in the probands, all 307 dogs acted as controls. Variants retained after the first stage of filtering, with the highest effect score and homozygous in both cases, were then further filtered against a VCF file containing 1987 WGSs consisting of 1611 dogs (321 breeds), 309 village dogs, 63 wolves and 4 coyotes curated by the Dog10K Consortium [16]. 

### 2.5. Sanger Sequencing of WGS Filtered Variants

Variants remaining after filtering that were homozygous in the PRA-affected ESDs were genotyped by Sanger sequencing in a small cohort of three additional PRA-affected and two PRA-unaffected ESDs (clinical status confirmed by veterinary ophthalmologists). Primers were designed using Primer3 [17] (Table S1) to flank the variant, which was amplified by PCR using HotstarTaq DNA Polymerase (Qiagen, Manchester, UK) with the following cycling conditions: 98 °C for 10 min; 35 cycles at 98 °C for 30 s; 57 °C for 30 s; 72 °C for 30 s; and then 72 °C for 5 min. Sanger sequencing was outsourced to Source Bioscience, Cambridge, UK, and PCR products were sequenced in both directions. Sequence traces were analysed using the Staden software package (v.2.0.0b11) [18]. 

### 2.6. SNP Genotyping and Homozygosity Mapping 

Wisdom Panel™ and Embark single nucleotide polymorphism (SNP) genotypic data from four PRA-affected and 22 control ESDs (control dogs were of unknown status not reported to be PRA-affected) were merged (86,808 SNPs) and assessed for runs of homozygosity in PRA cases using PLINK-1.9 [19]. Quality control of PLINK data included the exclusion of SNP variants with a minor allele frequency of <5% and missing genotype calls of >10%. 

ROHs in PRA-affected ESDs and absent in control dogs were then manually visualised in the Integrative Genomics Viewer (IGV) software (v.2.13.0) to search for structural variants that may have been underlying the association [20,21]. Any possible causal variants were then filtered manually by visualising genomes of other breeds within our in-house genome dataset (307 WGSs) and also with the Dog10k VCF file [16]. Structural variants which were homozygous in the ESD PRA-affected genomes and not homozygous, or heterozygous, in numerous control breeds were retained for further analysis.

### 2.7. SINE Insertion Variant Genotyping Using Sanger Sequencing and Amplified Fragment Length Polymorphism

An insertion/duplication located in an exon in *FAM161A* on chromosome 10 was investigated by Sanger sequencing of four PRA-affected and two control ESDs. PCR products were amplified using HotstarTaq DNA Polymerase (Qiagen, Manchester, UK) with the following cycling conditions: 98 °C for 10 min; 35 cycles at 98 °C for 30 s; 57 °C for 30 s; 72 °C for 45 s; and then 72 °C for 5 min. Primers were designed using Primer3 [17] (Table S2) to flank the insertion and produce a wild-type product size of 248 bp. 

Further validation of the variant was carried out using Amplified Fragment Length Polymorphism (AFLP) in six PRA-affected and 20 control ESDs (inclusive of 2 obligate carriers). A single FAM fluoresced tailed reverse primer was paired with wild-type and SINE-specific forward primers (Table S3). Amplified products were outsourced to the Department of Biochemistry, University of Cambridge, UK, for amplified fragment length polymorphism analysis using an ABI 3130×l DNA Analyzer (Applied Biosystems, California, USA). Fragment length analysis was then carried out using Genemarker v.3.0.1 (Softgenetics LLC, USA) with a wild-type fragment length of 266 bp and a SINE fragment length of 250 bp.

### 2.8. SINE Insertion Alignment

The ESD SINE located in *FAM161A* was aligned using Uniprot [22] against 25 canine-specific repetitive elements downloaded from Repbase [23] and the intronic *FAM161A* SINE sequence published by Downs and Mellersh [3] that is causal for PRA in Tibetan Terriers and Tibetan Spaniels.

## 3. Results

### 3.1. WGS Variant Analysis

Eighty-one previously published IED variants (including variants causal for neuronal ceroid lipofuscinosis) published in different canine breeds were excluded in the ESD probands after IED variant panel analysis, thus suggesting the presence of a novel PRA-associated variant segregating in the breed. The sire and dam of probands 1 and 2 (age 9 and 12 years, respectively) were confirmed to be clinically clear of inherited eye disease by a veterinary ophthalmologist. Therefore, WGS analysis was based on a recessive mode of inheritance, where control dogs had to be heterozygous or homozygous for the wild-type allele, and PRA-affected ESDa had to be homozygous for an alternate allele.

WGSs of 307 dogs (differing in health status and breed) acted as controls in the first stage of the variant filtering process. Results identified 13 variants specific to and homozygous in the probands with a predicted high effect on the protein. Eleven were subsequently excluded as they were common in the VCF data curated by the Dog10k consortium [16]. Two variants that were purported to be high-effect were private to the cases and located on chromosome 18 and within the genes *SPTBN2* and *SLC22A8.* Both genes were designated as possible candidates for PRA using the variant prioritisation software VarElect (v.5.19) with an overall score of 1.83 for *SPTBN2* and 0.99 for *SLC22A8* and a likelihood of causing disease average score of 64.48 and 47.0, respectively [24]. Both variants were missense variants, causing single-nucleotide changes of G > T in *SPTBN2* and G > C in *SLC22A8*. These were further investigated by genotyping additional breed-specific cases and controls. 

### 3.2. Segregation of Variants Identified through WGS Analysis of Cases

A cohort of five ESDs consisting of three PRA-cases and two controls (both obligate carriers and clear of inherited eye disease) were genotyped by Sanger sequencing for the two candidate variants in *SPTBN2* and *SLC22A8*. Neither variant segregated with the disease, with one PRA-affected ESD homozygous for the wild-type allele in both genes.

### 3.3. Runs of Homozygosity (ROHs) Detected in PRA-Affected ESD

**Figure 1:**
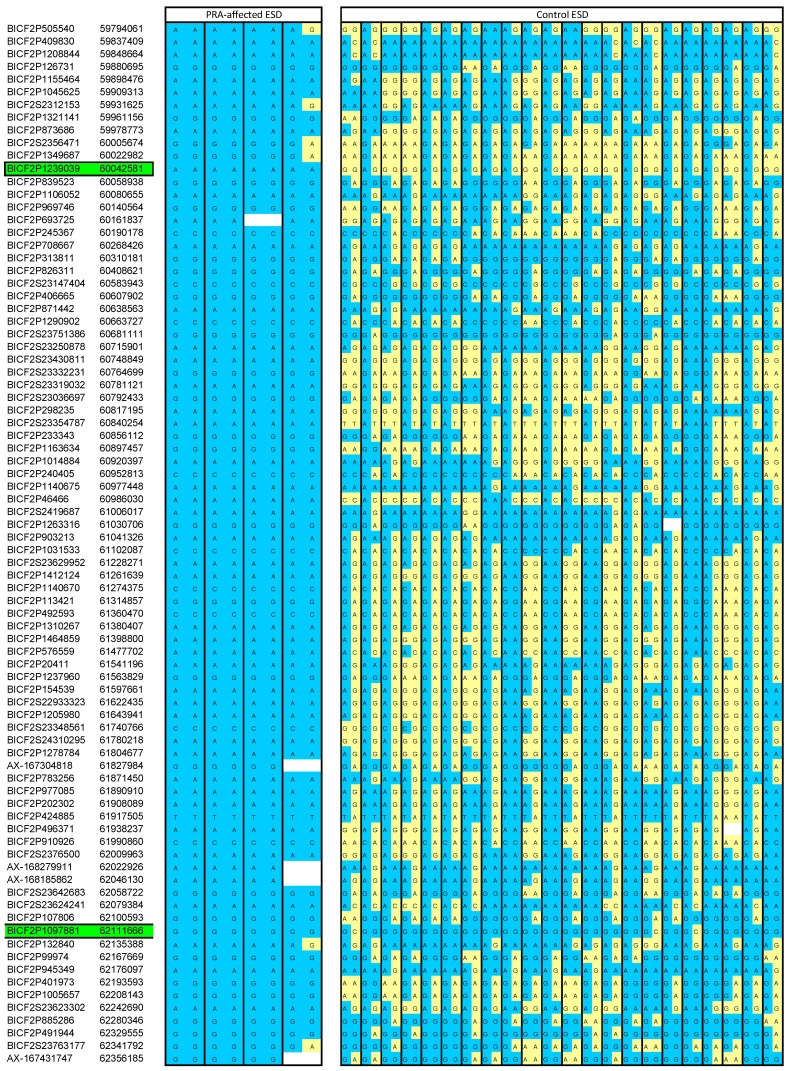
Homozygosity mapping of SNP markers to identify a critical region. A shared ROH was identified on chromosome 10, spanning approximately 2 Mb in all four PRA-affected ESDs and absent in the control ESD. The four cases are all homozygous for an alternative allele to the wild-type (indicated by blue cells). Yellow cells as shown in the control ESD cohort indicate a wild-type allele call. As the default parameters of an ROH analysis in PLINK 1.9 allow for one heterozygous call within the default scanning window, we were able to locate heterozygous calls and therefore narrow the region further to approximately 2 Mb. Green highlighted cells illustrate where the critical region commences and finishes (chr10: 60042581-62111666). All SNP marker co-ordinates are derived from the CanFam3.1 canine genome assembly. Blank cells indicate missing data.

The homozygosity analysis using available SNP array data revealed an overlapping ROH in all four PRA-affected ESDs on chromosome 10, which was absent in the controls. The ROH spanned approximately 3.8 Mb and further analysis enabled us to narrow the region further to approximately 2 Mb (Figure 1).

### 3.4. Identification of an Exonic SINE Insertion in FAM161A

**Figure 2:**
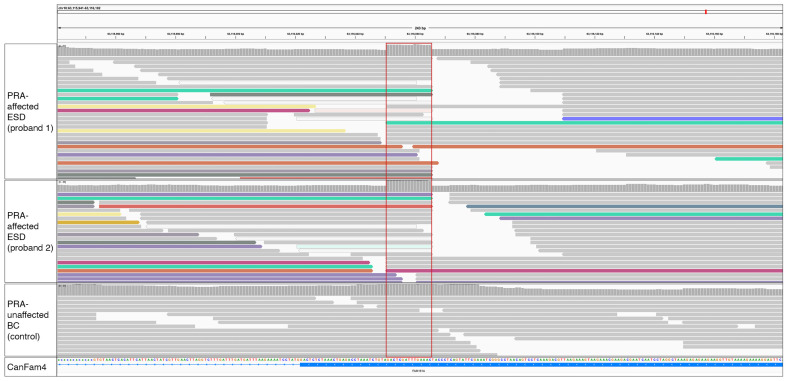
WGS reads over a 243 bp region of *FAM161A* inclusive of exon 4 of probands 1 and 2 and a control PRA-unaffected Border Collie dog. WGS reads are based on the canine reference genome UU_Cfam_GSD_1.0 (CanFam4). Alignments represent the two PRA-affected probands and a PRA-unaffected Border Collie that acted as a control. Alignments were coloured by ‘Insert-Size and Pair Orientation’ and reads surrounding the location of increased read depth varied in colour, indicating that read mates aligned to different chromosomes. The red-boxed area (chr10: 63,116,051 to chr10: 63,116,065) is located within exon 4 of *FAM161A* within the reference genome. The increased read depth is indicative of a duplication or insertion, absent in the control WGS. Grey reads are aligning to the reference genome and coloured reads indicate reads aligning to alternate chromosomes typical of a repetitive element insertion.

**Figure 3:**
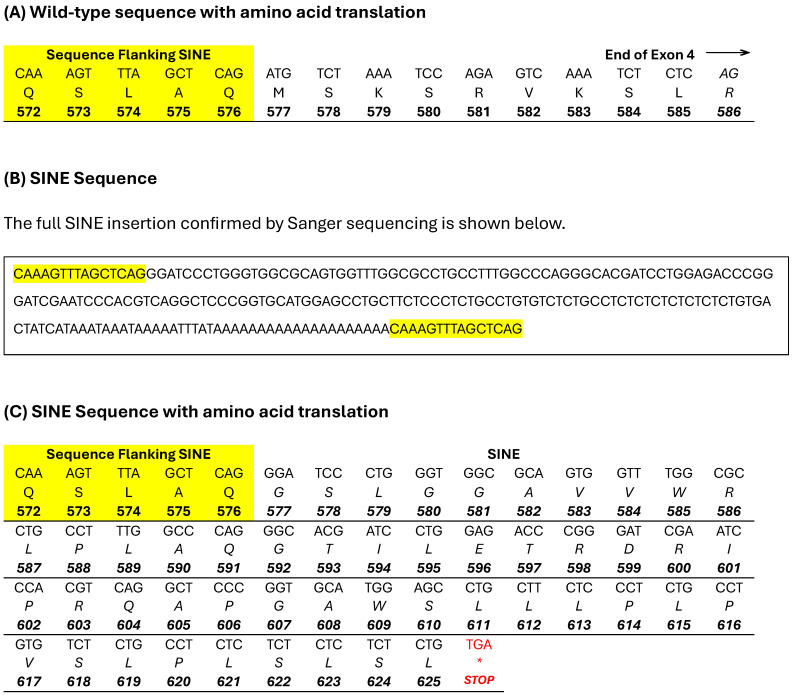
Wild-type and SINE insertion nucleotide and amino acid sequences. The wild-type sequence commencing at the flanking sequence (highlighted in yellow) of the SINE to the end of exon 4 of *FAM161A* is shown in (**A**). The top row is the nucleotide sequence, the second row is the amino acid translation and the bottom row is the amino acid position in the protein. (**B**) The full SINE sequence (210 bp) confirmed by Sanger sequencing is shown with the flanking sequence highlighted in yellow. (**C**) The SINE, again with the flanking sequence highlighted in yellow, is shown with the amino acid translation on the second row and the protein position on the bottom row. The SINE remains in frame and terminates with a stop codon (marked with an asterix in red), extending exon 4 by 39 amino acids compared to the wild-type sequence.

**Figure 4:**
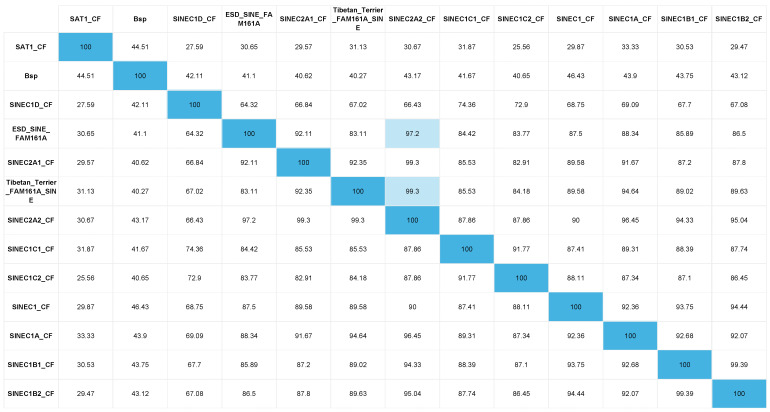
Percent identity matrix of canine repetitive elements. The nucleotide percentage identity between alignments of the ESD *FAM161A* SINE, the Tibetan Terrier/Tibetan Spaniel *FAM161A* SINE [3] and 10 canine repetitive elements [23] is shown in Figure 4. Shaded boxes illustrate the highest percentage identity identified between alignments.

The critical region identified in the ROH analysis was manually visualised using IGV for the presence of structural variants not detected by WGS pipeline analysis in the two probands. The WGSs of 307 dogs and the Dog10K VCF file [16] acted as controls to exclude common variants. This led to the identification of a structural variant located in the Family with Sequence Similarity 161 Member A (*FAM161A*) gene. This was the only variant identified that fitted the above filtering criteria and was homozygous in both proband WGSs and absent in all controls. A 15 bp region showing an increase in read depth was observed in exon 4, indicative of an insertion or duplication (Figure 2). By viewing alignments with the soft-clipped bases displayed, a poly-A tail was visible and therefore it was hypothesised that this was a transposable element (TE). The TE was confirmed by Sanger sequencing and by alignment with canine-specific repetitive elements to be a short interspersed nuclear element (SINE), 210 bp in size. The full sequence is illustrated in Figure 3B. The ESD SINE sequence shows the greatest nucleotide percentage identity (97.2%) with SINEC2A2_CF (Figure 4). 

**Figure 5:**
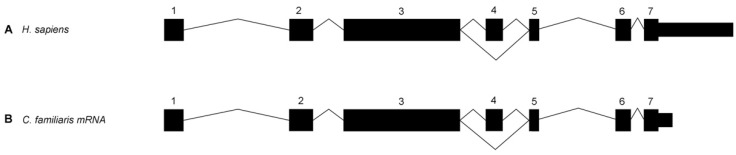
Exon–intron boundaries of human and canine *FAM161A.* Exon and intron boundaries are shown in human and canine *FAM161A*. Exon 4 is an alternatively spliced exon. This is modified from Downs and Mellersh (2014) [3].

**Figure 6:**
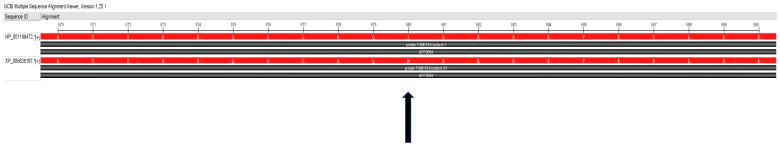
Human and canine FAM161A protein alignment. The protein alignment of the human (NP_001188472) and canine (XP_005626197.1) FAM161A protein is shown in Figure 6. The arrow indicates the position of the SINE identified in the PRA-affected ESD, located in the conserved UPF0564 region within exon 4. The alignment was carried out using the National Center for Biotechnology Information Constraint-based Multiple Alignment Tool version 1.25.0 [25].

The SINE commences at XP_005626197.1 c.17929/p.577 and is 210 bp in length. In the wild-type amino acid sequence, there are 10 amino acids that extend from the flanking sequence to the end of exon 4, as shown in Figure 3A. The presence of the SINE insertion after the flanking sequence is predicted to code for 49 aberrant amino acids, extending exon 4 by 39 amino acids, followed by a stop codon as shown in Figure 3C. The variant is located in an alternatively spliced exon which produces two *FAM161A* transcripts, a long and a short version which are both expressed in humans and in dogs (Figure 5) [3,8]. The SINE is located in the conserved UPF 0564 region of the protein, as shown in Figure 6. Validation of the SINE variant was carried out using amplified fragment length polymorphism in a total of six PRA-affected and 20 control ESDs, shown in Table 1. All PRA-affected ESDs were homozygous for the SINE insertion and controls were either heterozygous (*n* = 6) or homozygous (*n* = 14) for the wild-type reference. The dataset included the sire and dam of probands 1 and 2 and the grandsire/granddam on the dam’s side.

The SINE insertion variant was validated by amplified length polymorphism in 26 ESDs. The variant segregated consistently with a recessive mode of inheritance and the results are shown in Table 1.

## 4. Discussion

In this study, a combination of homozygosity mapping, using SNP array data and WGS, was used to identify a novel autosomal recessive variant that segregates within PRA-affected ESDs. WGS pipeline analysis of called variants (single-nucleotide variants and small insertions/deletions) in probands 1 and 2 proved insufficient in this study to provide a molecular diagnosis for PRA in this breed. Only 13 high-effect variants were homozygous in both WGSs, but variant filtering excluded 11 of these on the basis that they were common in multiple breeds. Although there are instances of inherited eye disease variants that are shared across dog breeds [3,26,27,28,29,30,31,32,33,34,35,36], they often have a shared lineage, and it is less common that they segregate across divergent breeds. Each canine breed can be considered as analogous to an isolated population, owing to centuries of selected breeding to fix desirable traits, and thus disease alleles are often breed-specific [37,38]. The two variants (both single-nucleotide variants) called in our pipeline analysis were located in the *SPTBN2* (Spectrin β and Non-Erythrocytic 2) and *SLC22A8* (Solute Carrier Family 22 Member 8) genes. Although neither of the genes have been associated with RP in humans according to Genecards [39], Malacards [39] or RetNet (https:// retnet.org, accessed on 20 May 2024), both are expressed in the retina [40,41] and so were flagged by VarElect [24] to be ‘directly related’ to the PRA phenotype, so they warranted further investigation. However, on further validation testing, neither variant segregated with the disease and both were excluded as causal for PRA in the ESD. This suggested that the causal variant may be of a structural nature.

The primary clinical sign of PRA in the dog is a loss of night vision. This is a consequence of rod photoreceptor cell degeneration that occurs in the initial stages of disease. Degeneration of cone photoreceptors then follows, resulting in a total loss of sight. An electroretinogram is required to ascertain whether a retinal disease is a rod–cone (as in PRA) or a cone–rod phenotype; however, this procedure is not routinely employed when assessing dogs. Owner observations of dog behaviour in dim light conditions are therefore a useful prognosticator for a PRA diagnosis. Night blindness was the primary clinical sign noticed by the owners of five of the six PRA-affected ESDs prior to a consultation with a veterinary ophthalmologist. Proband 2 was diagnosed during a BVA eye exam at age 4.9 years, having been reported to be unaffected at age 1.75 years, suggesting a late onset of disease. The median age of disease onset for PRA in the six ESD cases is 4.99 years, which is comparable to Tibetan Terrier and Tibetan Spaniels affected by PRA3, breeds in which the causal PRA variant is also located in the *FAM161A* gene [3]. There was one outlier in the ESD-affected cohort who was not diagnosed until almost 9 years of age. However, the owner reported that the dog had experienced difficulty manoeuvring in dim light and frequently bumped into objects for several months prior to diagnosis, so the true age of onset was likely earlier. An initial age of 8 years was chosen for ESDs to qualify as controls within the study. However, the ESD breed is numerically small and therefore obtaining suitable controls was challenging. Therefore, four dogs aged 7 years were included in the control cohort, all of whom were homozygous for the wild-type sequence. 

Our subsequent analysis of available SNP array data identified a single shared ROH present in four cases and absent in 22 controls, which was identified on chromosome 10 (see Figure 2). This was the only ROH that was present in all four PRA-affected ESDs. The region spanned just over 2 Mb and included 16 protein-coding genes (Supplementary File S2). The entire region, including intergenic and intronic regions, was manually interrogated in the two probands, using IGV. Candidate variants had to be homozygous in both cases and heterozygous in any other genome, but with the caveat that they were not common across multiple breeds. This approach yielded just one provocative candidate variant, an exonic short interspersed nuclear element (SINE) located in the Family with Sequence Similarity 161 Member A (*FAM161A*) gene. The SINE was homozygous and private to both probands. It was absent in the 307 control WGSs interrogated and in the Dog10K dataset [16]. This gene was first associated with RP in humans in 2010 [42] and with PRA in Tibetan Terriers and Tibetan Spaniels in 2014 [3], and was therefore deemed a strong candidate for PRA in the ESD. 

The *FAM161A* gene encodes a centrosomal protein that is expressed in various ciliated cell types and tissues [43,44]. In most tissue types, expression is low, with the exception of the testes, and in particular, the retina, where levels are high [42,45]. Expression in retinal tissue occurs in the photoreceptor inner segments, the inner and outer plexiform and ganglion cell layers [44,46,47]. The protein localises at the base of the connecting cilium, along the length of cytoplasmic microtubules and extends into the axenome of the outer segment of photoreceptors [48]. Evidently *FAM161A* is an integral component of photoreceptor cells; however, its functionality has remained somewhat of an enigma. This is further compounded by the fact that the gene produces two major isoforms, ‘long’, containing 7 exons (716 amino acids), and ‘short’, where exon 4 is spliced out (660 amino acids), with the significance of each unknown [3,8]. Only one domain within the protein has been identified to be evolutionarily conserved, yet is annotated to reflect the undetermined function as Uncharacterized Protein Family 0564 (UPF0564) [43,49]. Furthermore, despite the gene being expressed in different tissues, pathogenic mutations in *FAM161A* have to date effectuated an ocular-only phenotype in humans, PRA3-affected Tibetan Terriers and Tibetan Spaniels, and in the ESD PRA-affected dogs from this study.

**Figure 7:**
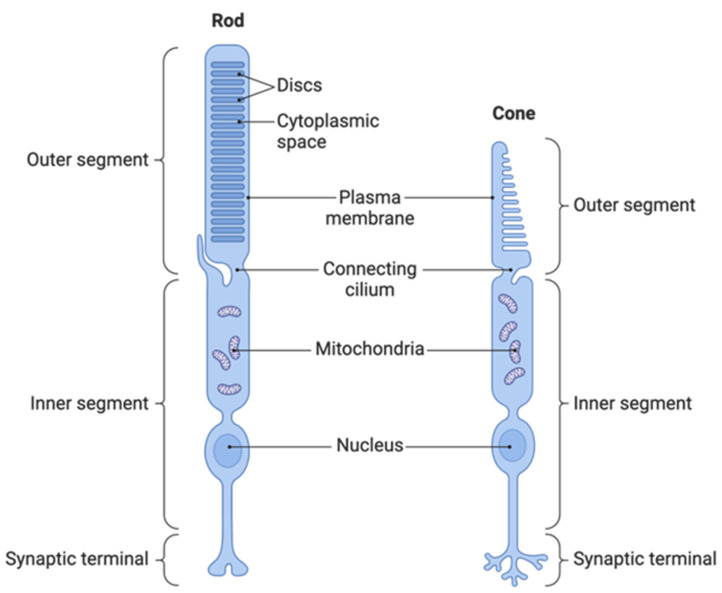
Anatomy of rod and cone photoreceptor cells. Reprinted from “Morphology of Photoreceptors” by BioRender.com (2024) retrieved from https://app.biorender.com/biorender-templates (accessed on 6 March 2024).

The works of Mercey and colleagues (2022) have proposed a compelling theory to explain *FAM161A*’s role in retinal disease. Splicing activity of retinal proteins occurs during retinogenesis, and the differential isoforms, including the long and short form of *FAM161A*, are largely targeted for use within the connecting cilium of photoreceptor cells [48]. The connecting cilium is a short but critically important bridge between the inner and outer segments of the photoreceptor cell (Figure 7). 

**Figure 8:**
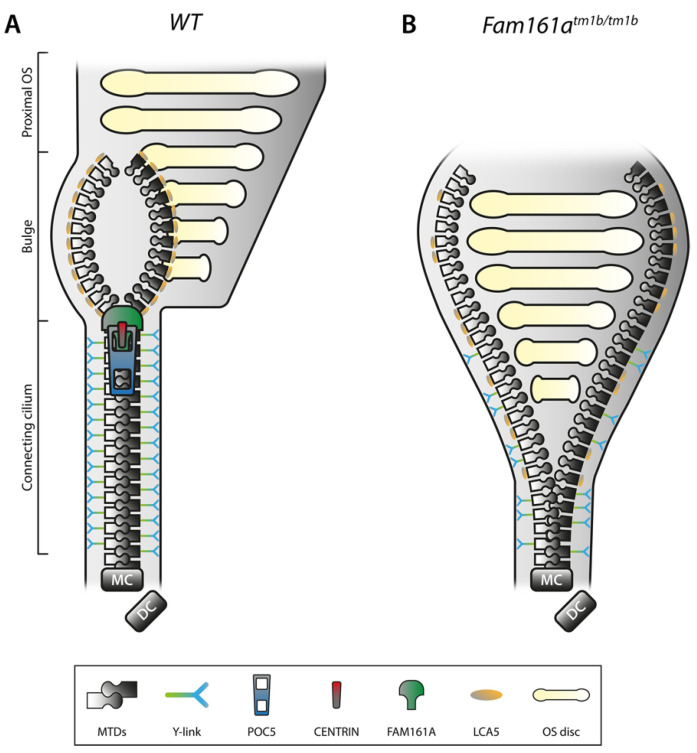
Schematic representation of the inner scaffold of the connecting cilium within a rod photoreceptor. Taken from Faber and Roepman (2022) [50]. (**A**) Schematic representation of a part of a WT rod photoreceptor consisting of the CC, the bulge region, and the proximal OS, including its membranous stacked discs. The MTDs are built up from the MC, accompanied by the DC. Cohesion of the MTDs in the CC is maintained by the inner scaffold proteins POC5, CENTRIN and FAM161A, located at the inner wall of the MTDs, comparable with a closed zipper. Note that these proteins are found all along the CC, in addition to the MC and DC. MTDs in the CC are connected to the membrane by Y-links, associated with CEP290 and SPATA7 localization. LCA5 localizes to the bulge region, where MTDs are more dispersed due to the absence of the inner scaffold and Y-links. (**B**) Deficiency of FAM161A causes loss of the entire zip head (the CC inner scaffold) as also POC5 and Centrin are absent, leading to spreading of the MTDs. This spreading, visualized by an open zipper, eventually causes a collapse of the OS structure. Protein localization at the Y-link level is secondarily affected when FAM161A is depleted, as seen by more dispersed CEP290 localization. Furthermore, FAM161A deficiency results in disorganization of the bulge region, obvious from LCA5 localizing more proximal to the MC. Altogether, the CC inner scaffold forms a structural foundation securing proper disc formation and OS integrity. DC, daughter centriole; CC, connecting cilium; MC, mother centriole; MTD, microtubule doublet; OS, outer segment; and WT, wild-type. *FAM161A^tm1b/tm1b^* is mouse model where exon 3 is knocked out, resulting in a short (only exons 1 and 2) non-functioning protein [51].

It is via this route that synthesised proteins are trafficked from the inner to the outer segments of the photoreceptor to be utilised for either phototransduction or for outer segment formation [44]. Mercey and colleagues (2022) found that FAM161A, in conjunction with POC5 and CENTRIN, form an ultrastructure within the connecting cilium, described as a “structural zipper” that maintains the integrity of the microtubule doublets within the core of the connecting cilium (see Figure 8) [44]. In *FAM161A*-deficient mice, cohesion of the microtubule doublets becomes progressively impaired, causing them to spread, which induces the collapse of the inner scaffold and culminates in the total collapse of the photoreceptor outer segment and thus photoreceptor cell death. However, loss of FAM161A within the centriole inner scaffold, a structure similar to the connecting cilium inner scaffold, does not result in the same outcome. It was found that POC5 and CENTRIN remain despite the absence of FAM161A and are presumably still able to function. This suggests an extremely specialised role for *FAM161A* within this ultrastructure and may explain how its loss or malfunction does not impact other tissue types. 

PRA is intractable to treatment and therefore veterinary care of affected dogs is focused on monitoring disease progression. Surgical intervention may be required if severe cataracts develop, which can occur as a secondary complication to PRA [52]. This was not the case for any of the ESD PRA cases included in this study and therefore tissue was not available with which to carry out any RNA analyses. For that reason, we can only postulate how the c.17929ins210 SINE might be causing PRA in this breed of dog. SINE insertions are present in the canine genome in high numbers and can exert a positive influence on gene functionality [53,54]. There are, however, inherited diseases that have been reported to be caused by SINE insertions, including an intronic SINE in *FAM161A* that is associated with PRA in Tibetan Terriers and Tibetan Spaniels and an exonic SINE in *STK38L* that causes early retinal degeneration in Norwegian Eklhounds [5,55,56]. The ESD and Tibetan Terrier/Tibetan Spaniel *FAM161A* SINEs both share a high percentage of identity with the SINEC2A2_CF SINE (97.2% and 99.3%, respectively), which is known to be highly active in the canine genome [57]. It is intriguing that two disease-associated SINE’s have been identified in this gene in the three dog breeds, and within close proximity of one another. It is postulated that as SINEs are nonautonamous, their amplification may be contingent on Long Interspersed Nuclear Elements (LINEs) [58]. According to the UU_Cfam_GSD_1.0 (CanFam4) genome assembly, there are two LINEs (L1MA9 and L2c) that flank exon 3 and exon 5 of *FAM161A* (Figure S1). It is possible that the presence of the two LINEs allows for the proliferation of SINEs within this region, two of which are annotated in the reference assembly (SINEC_Cf and MIR3). 

The fourth exon of *FAM161A* is only included in the longer form of the protein and therefore the SINE insertion identified in this research would only impact that isoform. Downs and Mellersh (2014) found that, as in humans, expression of the long isoform of *FAM161A* is much lower than the short form [3,8]. The long isoform is concentrated mostly in the retina and is rare in other tissues [48], which intimates a vital and specialised role in this region of the central nervous system. Exon four of *FAM161A* is located in the terminal region of UPF0564 and this conserved region is predicated to contain two coiled-coil domains [43], which Levine (2020) postulates are homologous to regions present within the microtubule nucleation factor gene, *TPX2* [49]. The c.17929ins210 SINE would in the first instance produce an erroneous transcript at the terminal region of exon 4, which, in the wild-type transcript, relates to the last 10 amino acids of UPF0564, but furthermore cause termination of protein translation as a consequence of a stop codon at the aberrant p.626 position (Figure 3C). The sequence homology postulated between *TPX2* and *FAM161A* and the “structural zipper” role proposed by Mercy and colleagues (2022) both indicate that *FAM161A* possesses a vital association with microtubules, and specifically within the connecting cilium. As the C-terminal of the protein is hypothesised here to be missing, inclusive of the terminal section of UPF0564 containing coiled-coil domains, it is feasible that this essential functionality is impaired. In the human, pathogenic variants in *FAM161A* causal for RP are predominantly located in either exon 3 or exon 5 [8], which can potentially disrupt the functionality of both the long and short isoforms of the gene. RP pathogenic variants in exon 4, as observed in the ESD cases in this study, are more rare in humans; however, in the limited cases published, the variants have been in the form of introduced stop codons, which in one case that was investigated, resulted in early termination of the protein [59,60]. In Tibetan Terriers/Spaniels affected with PRA3, a *FAM161A* c.1758-15_1758-16ins238 SINE insertion causes aberrant splicing out of exon 5, and thus could also potentially impact both isoforms [3]. Interestingly, the mutated isoform was detected in the blood of both an unaffected and a PRA-affected dog; however, the latter had markedly increased levels in comparison to the unaffected control, particularly in relation to the long mRNA transcript. All of the aforementioned studies indicate that pathogenic genetic variants in the dog and in humans that either disrupt the balance of the expression of the short and long isoforms of *FAM161A*, or produce a null form of the protein, all result in the retinal diseases of RP and the canine-equivalent disease, PRA. The role of the gene appears to be one of photoreceptor maintenance, as both cell types are able to form in spite of damaging alterations in the *FAM161A* gene. This was observed in the ESD PRA cases whose diagnoses did not occur until a late stage. Furthermore, we postulate that only the long isoform is negatively impacted in the breed, which illustrates that the short form is unable to maintain cellular health in isolation. We hypothesize that the PRA-associated variant in the ESD is specific to the breed and would therefore not be applicable as a variant specific model for gene therapy in humans. However, the location of the variant does provide information as to the importance of the longer isoform in ocular health and may therefore benefit ongoing and future gene therapies. This is corroborated by *FAM16A* adeno-associated virus gene therapy, where applications using the longer isoform have achieved the most propitious results in mouse retinas [8]. 

In summary, our study investigating PRA in the ESD has identified an exonic SINE in the alternatively spliced exon 4 of *FAM161A*, where variants are rare in RP-affected humans. Although no definitive function has been identified for either isoform of the *FAM161A* gene, it appears evident that the longer form is essential for photoreceptor maintenance and survival. Although functional analysis of the effects that the SINE insertion exerts on the protein was not possible in this study, an aberrantly introduced stop codon in exon 4 identified in an RP-affected human caused truncation of the protein [60]. The age of disease onset of PRA in the ESD is equivalent to that observed in Tibetan Terriers/Spaniels, suggesting that maintenance of the photoreceptor cells is negatively impacted by the gene’s genetic alteration, which may be as a consequence of connecting cilium collapse. The variant was fully penetrant in the six PRA cases and is seemingly limited to the ESD breed. A DNA test designated PRA6 is now available for ESD breeders/owners. This is deemed to be an invaluable breeding tool as carriers of the variant can now be easily identified, allowing them to be retained in the breeding pool, and therefore maintaining genetic diversity in this small canine breed. Since the launch of PRA6, 47 ESDs have been tested for the variant, which has been identified in populations located in both the UK and the US with an allele frequency of 0.28. Finally, the dog may once more act as a model for human disease and associated gene therapy research.

## Figures and Tables

**Table 1:** Results of amplified fragment length polymorphism in PRA-affected and control ESDs.

Disease Status	Homozygous SINE +/+	Heterozygous SINE +/-	Homozygous Wild-Type -/-	Total
PRA-Affected	6	0	0	6
Clear	0	6	14	20

## Data Availability

The original data presented in the study are openly available in the European Nucleotide Archive (ENA) at EMBL-EBI under accession number: PRJEB75721 (https://www.ebi.ac.uk/ena/browser/view/PRJEB75721 (accessed on 18 July 2024). The BioSample accession numbers are as follows: SAMEA11296803 and SAMEA11296823.

## Supplementary Materials

The following supporting information can be downloaded at: https://www.mdpi.com/article/10.3390/genes15070952/s1. File S1. Gel electrophoresis and amplicon array panel of IED variants; Figure S1. Illustration of exons 3 to 5 of FAM161A; Table S1. Primers for WGS filtered variants; File S2. Genes in the shared run of homozygosity region detected in four PRA-affected ESD; Table S2. Primers used to Sanger sequence *FAM161A* SINE insertion; Table S3. Amplified fragment length polymorphism primers for *FAM161A* SINE identification.
