# Supplementary material for: Exonic Short Interspersed Nuclear Element Insertion in FAM161A Is Associated with Autosomal Recessive Progressive Retinal Atrophy in the English Shepherd
Source: Genes (Basel). 2024 Jul 20;15(7):952. doi: 10.3390/genes15070952 (PMC11275866; doi:10.3390/genes15070952)
Supplement: Supplementary file 1 [file genes-15-00952-s001.zip › File S1.pdf]

File S1. Gel electrophoresis and amplicon array panel of IED variants

Massively parallel sequencing was carried out using the following protocol.

- Variant amplicon primers were combined to create three primer mixes containing a maximum of 60 primers.
- Amplicons were amplified by 2-step PCR using HotstarTaq DNA Polymerase (Qiagen) and $MgCL_2$ at a concentration of 1.00mM, primer mix at a concentration of 0.10µM with the following cycling conditions: 95°C for 10 mins; 25 cycles at 95°C for 30s; 60°C for 4 mins. Two amplicons marked with an asterix below were run in a separate PCR amplification using HotstarTaq DNA Polymerase (Qiagen): 98°C for 10 mins; 35 cycles at 98°C for 30s; 61°C for 30s; 72°C for 30s and 72°C for 5 mins.
- 10µl of each PCR amplification was combined and 40µl (per sample) was purified using a ratio of 1.5x Ampure XP Beads (Beckman Coulter).
- An NEB Next Ultra II 150bp paired end library was prepared, using NEB Next multiplex oligos for Illumina, and sequenced on an Illumina Miseq. Paired end sequence data were aligned to the CanFam3.1 canine reference genome.

Amplicon gel electrophoresis.

- Amplicons for ADAM9 were amplified using PrimeStar GXL polymerase (Takara) with the following cycling conditions: 30 cycles at 98°C for 10s; 60°C for 15s; 68°C for 2 mins.
- Amplicons for PPT1 (gel electrophoresis) were amplified using HotstarTaq DNA Polymerase (Qiagen): 98°C for 10 mins; 35 cycles at 98°C for 30s; 57°C for 30s; 72°C for 30s and 72°C for 5 mins.

Gene	Variant	Fragment Length	Forward Primer	Reverse Primer
ADAMTS10	p.G661R	200	GTGGAAGTGGGAGTGTCAATG	TGAAGGCCTGTTCACTCACAT
ADAMTS10	c.1441G>A	254	GAAAGGGTTGGTCTTCATGGT	CTGCAGCATTAACGAGGACAT
ADAMTS17	c.1473+1G>A	224	ACTACAGTGCCAACGAGCAGT	AACTGCCTGTGAGGGTGTATG
ADAMTS17	c.193_212_del	238	GCTGACGCGTCTCCTCTCT	CGAAGCTGCAGGTACAGGTC
ADAMTS17	c.1552G>A	283	TCTGACCCCAGTAGTGACCTG	AGCAGCCAGTGGAGCTTTAG
ADAMTS17	c.3069_3074del6bp	287	AGCCTCCTTGTCCTGCATTAT	TCTTGTCATTGCAGACCTCCT
BBS4	c.58A>T	212	GCAAGATACATGGTGTTGCTTT	GCTTTCCCCAAAAACTCACAT
BEST1	c.73C>T	299	TGAATGGCTGGCTATTTGTTC	AGCCCCCTATCAGTGTACCTC
BEST1	c.482G>A	299	ACGCCTTCATTGACAGGTTG	CCTCACGTGGTTCTGATGAGT
BEST1	c.1388del1bp	276	TTATCCCAGGCTGTAGGCTTT	AAGAAGGCTTTCTCCAAGACG
C17H2ORF71*	c.3149_3150insC	243	GAGGGGGAACTGGCCTCT	CACAAGAGGCTCCTGGACAC
CCDC66	c.521_522insA	253	TTCATCCAGGACAGATGAAAGA	CGTAATTCCCACTGAAGGACA
CHST6	c.814C>A	282	CAGGTGCTCTACTCGCTGCT	ATCCTCGAAACGCACCAG
CNGA1	c1752_1755delAACT	237	TTCAGTTTCTGCTGCATTGAC	AAAAGATGACCTCATGGAAGC
CNGA3	c.1270C>T	293	ATCTCGGCCTTCAATTTGTCT	CCCCCTGTAAAAGATGAGGAG
CNGA3	c.1931_1933delTGG	223	TTTCACTTTCATCTGGGTTGC	GGAGGCTCTCACTGAATACCC
CNGB1	c.2685delA2687_2688insTAGCTA*	293	GAGTTGACAACAAGCCTCTGC	AATAGTTCAGCCCCTGGAAGA
CNGB3	c.784G>A	253	CAGCCCCACTTACCATTATGT	ATGCTTGTTACCTGCCATTTG
COL9A3*	g.49699847insG	245	AGGTGGGCCCGGCTGAAT	CGAGGCCCGCTCACATGC
FAM161A	c.1758-15_1758-16ins238	274	TTCATCTCTTCAGGCCAGTGT	AAACAGTAGCGGCCTGTTTTT
FAM83H	c.1015delC	280	GTCGAGGAAAGAGGGGAATC	CATGTGGTCCTTCGAGAAGAT
HSF4	g.85286582_3insC	277	TCATGAACTCTTGGCTCACCT	GGGTCTGGTTGAAGCTTTTCT
HSF4	g.85286582delC	277	TCATGAACTCTTGGCTCACCT	GGGTCTGGTTGAAGCTTTTCT
IQCB1	c.952-953insC	278	GAGGGAAAAAGCACCCTAAGTT	TGCCCAGGCCTCATATTTTAT
OLFML3	c.590G>A	210	AGATGGGACACAGAACGACAC	ATGAGTTGCAACGTGTTCTCC
PDE6B	c.2420G>A	205	AGACCTTTCTTGGCTGTCGTC	GAACTGTCGAGAGGTGTGGTC
PDE6B	c.2448_2449insTGAAGTCC	205	AGACCTTTCTTGGCTGTCGTC	GAACTGTCGAGAGGTGTGGTC
PDE6B	g.91763017G>A	275	GATCATTCACTGGGGGAAAAG	TTACCTTTGATGCACGGAAGT
PDE6B	c.2404_2406delAAC	205	AGACCTTTCTTGGCTGTCGTC	GAACTGTCGAGAGGTGTGGTC
PDE6A	c.1940delA	216	TATTTCTGGCCTTCAGCCTTT	TGGTGTCTTTCCAAGATGGAG
PRCD	c.G115G>A	259	ACCCCCTAGCTTCTCCTCTCT	AGCTTGAGCCTCCTAATCCAG
RHO	c. 11C>G	242	GTGACGTAGAGCGTGAGGAAG	CCTCAGCAGCACTCTTAGGAC
RBP4	c.282_284del	253	ACGATGTTGTCCTGCAGAAAG	GACTGCCGAGTGAGCAACTT
RPE65	c.487_490delAAGA	242	TTACTTCCGAGGAGTGGAGGT	GCAGCGAGACAATCTATTTGG
RPGRIP1	g.8228_8229insA$_{29}$GGAAGCAACAGGAT	221	GTGAAGAGCACATGTTGGTGA	GTTGAGCTTTGTTTGCCTTTG
SAG	c.1216T>C	261	ATCCTGATTGGTCCCTCTGTT	CCTGCGTGAACTACTGTTTCC
SLC45A2	c.1287delC	271	ACCGCCAGCTGTAATTTCTTT	GGGTGAGGTTCAATGACACAC
SLC45A2	c.1487G>A	279	CAGAAAAAGTGTGCCAGCTTC	AAAGAGAGCCACAAAACAGCA
SLC4A3	c.2601_2602insC	262	CAGGTACGTGAGGGGTCAGT	ATGAGGATGAGGGACAGCAG
STK38L	c.20447905_20447906ins315bp	293	CAGTGTTGCAATTTGCCATT	GAAAAAGTTACGCCGATCACA
TTC8	c.669delA	270	CATCTGGAAACATGAGCCACT	CCATGTCTAAGCCCTTCACAA
ABCA4	c.4176insC	273	CATCTTGCCTTTTCACGTCTC	ATACATCCAGGGGTGAAGGAT
OCA2	c.377+2T>G	203	CGGAGGAGCTCACACTGC	GCTTTCCGCTCAGTGGTC
NPHP4	g.59912988del180bp	253	AACCATCCAAGCTCATGACAC	TTTGCAGGTTGAGGCTGTATC
NECAP1	c.544G>A	249	GGCTTCAAGGAAGGACAGACT	TAGCAACTGAGGAGGATGGTG
IMPG2	g.7785475_LINE_Insertion	284	GAACACACCAAAAGGCTTGAA	GCCAGTTAAAGGCAAAGAACC
PPT1	c.736_737insC	207	ACTCTTTGCTGCAGCTTTTTG	CTCCCCTCTGGTTACCTGTGT
PPT1	c.124+1G>A.	208	GTGATCGGGCCAAGATGG	CAGACGCGAACCCAGGTC
TPP1	c.325delC	288	GAGTGACTCGTCTCCCTGATG	AAGTCCCAATGTCTCCTGGTT
CLN5	c.619C>T	253	AAATGGCAAAGTGGGTAAAGC	GGCCCAAAAATAGAGGTTTCA
CLN5	c.934_935delAG	292	GGCCGACAGGAAATAAGACTC	TATGCTGGTGACAGAAGGAAA
CLN6	c.829T>C	200	ATGACACCCGGGTACTTCTTC	GGCCAGATCTTCATCCTCTTC
MFSD8	c.843delT	266	TGCTTTGAAGCCTAAAAGTGATT	TTACGTTTCAAAAAGGGCAAA
CLN8	c.491T>C	245	AACTGGTGCTGGTTTCACATC	ACCTTTAGGAGCATCCAGGAA
CLN8	c.585G>A	223	CTGGTCCGAGTCTCTGTTTTG	AAGCTGCTGCGTCTTCTTATG
CTSD	c.597G>A	249	CTTTTGCTGCATCAGGTTGTC	GGGTCCTCTGTCTTGAGTCCT
ATP13A2	c.1620delG	253	GGTGTGGTGCCCCTAAAAG	GAAGTCCTGGAATGAAGACCA
ATP13A2	c.1118C>T	295	TGCCTGCATGTATGGTTGTAA	CCACCTGTCTGAGTCACCACT
ARSG	c.296G>A	297	AACACTGGAATCCTGGGAACT	AGCATCGGGGTTACCTATCAT
CLN8	c.349insT	245	AACTGGTGCTGGTTTCACATC	ACCTTTAGGAGCATCCAGGAA
COL9A3	c.700C>T	296	AGGTGTTCCTGGGACGACTG	AGAGGCCCCTAACCCTCCAT
SIX6	c.487C>T	272	GGTGGACAAGTACCGTGTGAG	AGGCGTGTAGGCCTCTAGGTA
LRIT3	c.762_763delG	261	GGTTTAGGTCTGCAGGACAAC	AGGGTAGCCAGTAGCATCACA
PPT1	g.2,872,023delAG and g.2,872,103G >	246	CATTACAGGGGTTTTGTTTGC	GCTGCATCTGACACACATGAT

Gene	Variant	Wild Type Product (WT)/Mutant Product (MUT)	Fragment Length	Forward Primer	Reverse Primer
ADAMTS17	chr3:40812274 / chr3:45768123 Inversi	WT	293	TTCCATCATTAGAGGGAGCTG	CAAAGCCTTTCCATGTCAGTC
		MT	308		TTCAAAATAGGTATCTATGGAGGTG
SLC45A2	chr4:73867274 - 4081bp Deletion	WT	254	CACTGACTCGTTTATCCACTGC	ACCATGCAGGTGAGGACTG
		MT	281		CGTCGGTGACTGAGCAGA
CNGB3	29:32695888 404820bp deletion	WT	209	CTATTTCAGTGGGACCGTATG	CACTGTTGACCACTCGTATTC
			245		GAGGTAGAAGTTTGCCCAAGA
COL9A2	2647670 1267bp deletion	WT	208	CAAATTCAGCTCCAGGAACAC	AGGGGCTTCATAAATTTCAGC
		MUT	286		GTGAATGGGCACCATTGTCT
MERTK	chr17:36338043 LINE Insertion	WT	289	GAATAAACACATCCCTGGCATT	GCAACTTAATGTGTGCTGCTTA
		MUT	228		CTACTCTTCCGCCATCTTGCT
NHEJ1	c.588+462_588+8260del7799bp*	WT	268	GTTCTCCAGCCAAAACTTTCC	ATAACTCGAGGCCAGGTTGTT
		MUT	295		GTGAAAAGCACAGGGTTACCA
CLN8	g.30852988_30902901del	WT	219	GGTAATGCCACTGTTGACCAC	TCCATGGGTTTGTTCACTTTC
		MUT	207		AGTACAACTAGCATGTCAAGCTCAA
STK38L	ins20447905_20447906_ins15bp	WT	289	ACCTCTCCAAATGCTCCTCTT	TTTCCAAGCTTACCAAATACTCAAC
		MUT	229		GGATCGAATCCCACGTCA

Gene	Variant	Fragment Length	Forward Primer	Reverse Primer	
PPT1	chf15:2874608_2875553	538	GCATCCACTTAGGCACGTTT	GAACACACACTGGGCTTTCC	Gel Electrophoresis
ADAM9	23221bp del	WT (0) / MUT 1,515 bp	CTGGAGCAATGGGGCTGGATA	TCAAAGGAGCAATCGGAAAAGTCT	Gel Electrophoresis
