# Supplementary material for: Exonic Short Interspersed Nuclear Element Insertion in FAM161A Is Associated with Autosomal Recessive Progressive Retinal Atrophy in the English Shepherd
Source: Genes (Basel). 2024 Jul 20;15(7):952. doi: 10.3390/genes15070952 (PMC11275866; doi:10.3390/genes15070952)
Supplement: Supplementary file 1 [file genes-15-00952-s001.zip › Table S1.pdf]

Gene	Forward Primer	Reverse Primer	Amplicon size (bp)
SPTBN2	GGGGCAGGACTCTATGGAATA	GACCTCCTTTCTGCACAGTTG	432
SLC22A8	TGGATACCAGAGTCCATACGC	GCGACACTATACTTGGCCTTG	371

Table S1. Primers for WGS filtered variants
