# Supplementary material for: Exonic Short Interspersed Nuclear Element Insertion in FAM161A Is Associated with Autosomal Recessive Progressive Retinal Atrophy in the English Shepherd
Source: Genes (Basel). 2024 Jul 20;15(7):952. doi: 10.3390/genes15070952 (PMC11275866; doi:10.3390/genes15070952)
Supplement: Supplementary file 1 [file genes-15-00952-s001.zip › Table S2.pdf]

Forward Primer	Reverse Primer	Wild-type product size (bp)	SINE insertion product size (bp)
TGCACATTGAGTGTAAGTAATTGA	CTTAGTTTGTTGCCCTCAGGA	258	468

Table S2. Primers used to Sanger sequence *FAM161A* SINE insertion.
