# Supplementary material for: Exonic Short Interspersed Nuclear Element Insertion in FAM161A Is Associated with Autosomal Recessive Progressive Retinal Atrophy in the English Shepherd
Source: Genes (Basel). 2024 Jul 20;15(7):952. doi: 10.3390/genes15070952 (PMC11275866; doi:10.3390/genes15070952)
Supplement: Supplementary file 1 [file genes-15-00952-s001.zip › Table S3.pdf]

Tailed reverse primer	TGACCGGCAGCAAAATTGGTTGCCCTCAGGAGATCACT
Wild-type forward primer	TGCACATTGAGTGTAAGTAATTGA
SINE specific forward primer	CTGACGTGGGATTCGATC
FAM fluoresced primer	TGACCGGCAGCAAAATTG

Table S3. Amplified fragment length polymorphism primers for *FAM161A* SINE identification
