# Supplementary material for: Exonic Short Interspersed Nuclear Element Insertion in FAM161A Is Associated with Autosomal Recessive Progressive Retinal Atrophy in the English Shepherd
Source: Genes (Basel). 2024 Jul 20;15(7):952. doi: 10.3390/genes15070952 (PMC11275866; doi:10.3390/genes15070952)
Supplement: Supplementary file 1 [file Figure S1.pdf › Figure S1.pdf]

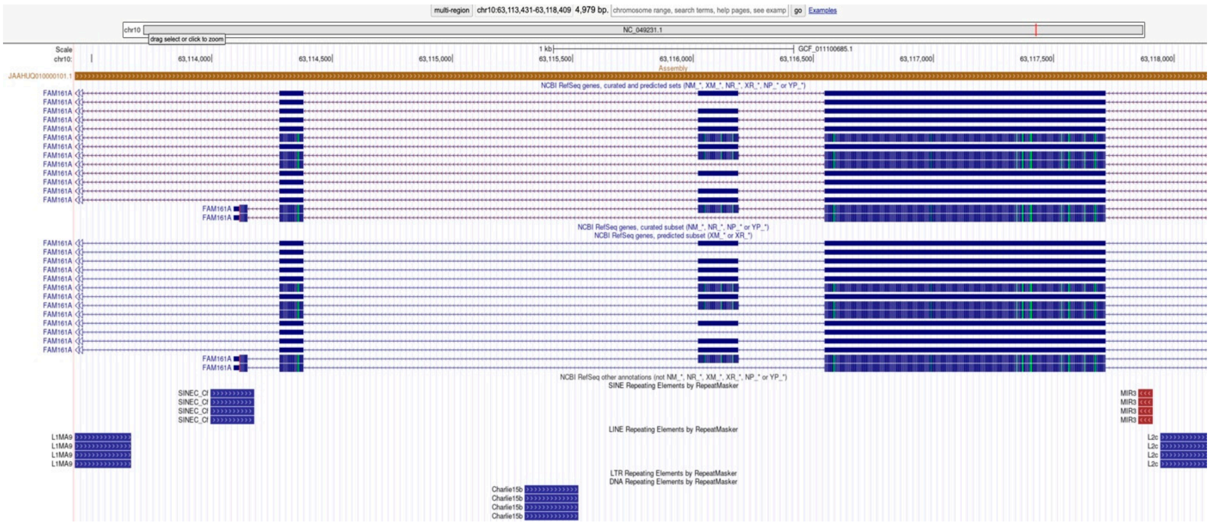

Figure S1. Illustration of exons 3 to 5 of FAM161A

Two LINEs flank exon 3 and exon 5 in FAM161A annotated as L1MA9 and L2c. Additionally two SINEs are present within the region.
